# From trichophagia to trichobezoar: Rapunzel syndrome in a child – A case report and literature review

**DOI:** 10.1016/j.ijscr.2025.111940

**Published:** 2025-09-15

**Authors:** Umer Iqbal, Sara Jawaid, Sarah Sohail, Syed Ali Arsal, Rehman Asif, Inibehe Ime Okon

**Affiliations:** a Department of Medicine, Shaheed Mohtarma Benazir Bhutto Medical College, Lyari Hospital Rd, Rangiwara Karachi, Karachi, 75010, Pakistan; b Department of Medicine, Jinnah Sindh Medical University, Rafiqui H.J, Iqbal Shaheed Rd, Karachi Cantonment Karachi, Karachi City, Sindh, 75510, Pakistan; c Department of Research, Medical Research Circle (MedReC), Bukavu, DR, Congo

**Keywords:** Gastric trichobezoars, Rapunzel syndrome, Gastric mass, Case report, Literature review

## Abstract

**Background:**

Gastric trichobezoars are suggestive of Rapunzel Syndrome, a rare condition typically affecting young women with underlying psychosis. Patients show trichotillomania and trichophagia, which, over time, produce a stomach trichobezoar. This mass may develop a characteristic tail-like protrusion in the small intestine.

**Relevance:**

Rapunzel Syndrome should be considered in the differential diagnosis, given its rarity and variations in symptoms among individuals. Early identification helps to avoid misdiagnosis, aggravation of the illness, and inadequate therapy plans.

**Case presentation:**

A 7-year-old female patient presented with a severe stomachache, weight loss, and trichophagia. A physical examination revealed a lump in the epigastric area, an intragastric mass, and mild hepatomegaly. Jejunojejunal intussusception with mesenteric lymphadenopathy was verified with a contrast-enhanced abdominal CT scan, which is a key unique presentation of this case. A successful laparotomy was performed, and a wound infection with E.coli and Candida species after surgery was treated with Amikacin and dressing.

**Discussion:**

From being asymptomatic to generating major difficulties, Rapunzel Syndrome shows a wide spectrum of symptoms. This disorder causes bacterial or fungal infections in many people; hence, recurrence is often seen in those who neglect mental consultations. Treatment plans differ; endoscopy is appropriate for some situations, but more severe cases call for surgical intervention.

**Conclusion:**

Trichobezoar is an unusual illness that, if not identified on time, can result in serious medical complications. An accurate diagnosis depends on a complete examination, appropriate history-taking, and early investigations. To prevent relapse, psychiatric follow-up is crucial, and laparotomy is still the gold standard technique.

## Highlights

• Rapunzel Syndrome is a rare condition involving hair masses in the stomach extending into the intestines, mostly seen in young females with psychiatric illnesses like trichotillomania and trichophagia.
• Symptoms like abdominal mass and weight loss in young girls should raise suspicion for trichobezoars and Rapunzel Syndrome.
• Ultrasound and CT scans are valuable tools for diagnosing gastrointestinal bezoars and complications like intussusception.
• In cases of large trichobezoars requires surgical removal via open or endoscopic approach
• Collaboration between pediatricians, surgeons, and mental health professionals ensures comprehensive care and reduces the risk of recurrence.

## 1 Introduction

An indigestible mass that builds up in the gastrointestinal (GI) tract and solidifies into a mass is called a bezoar [1]. Both food-based and non-food ingredients can take up this form. “Bezoar” comes from the Arabic word “Badzehr” or the Persian word “Padzhar,” which means “antidote” [2]. Based on their composition, bezoars are majorly divided into four types: phytobezoars, which are the most prevalent and are made up of indigestible fibers from fruits and vegetables [1]; trichobezoars, which are formed when hair accumulates, are typically black regardless of the patient's natural hair color due to action of stomach acid [3]; pharmacobezoars, which are composed of undigested medications [3]; and lactobezoars, which are more frequently found in infants and consist of undigested milk and mucus [4]. Furthermore, not all bezoars fit into these clearly defined categories and thus, are referred to as “other types of bezoars”. Including those made up of materials such as sponge, paper, plastic, or chewing gum [4]. Geographical location, cultural customs, and socioeconomic status all have a substantial impact on eating preferences, and the prevalence of bezoars varies accordingly.

Until they become large enough to cause distress resulting in severe consequences such as intussusception, perforation, appendicitis, and peritonitis, bezoars frequently show no symptoms, remaining unnoticed for a long period [5].

One rare form of trichobezoar is Rapunzel Syndrome. It occurs when a stomach bezoar spreads widely, extending like a tail into the small intestine [6]. Although it can occur at any age, females are far more likely to experience this illness, especially if they have developmental delays or psychological issues [7]. Trichobezoars are closely associated with trichotillomania, a disorder characterized by excessive hair pulling, and trichophagia, the compulsive ingestion of hair [8]. This work has been reported according to the Surgical Case Report (SCARE) 2025 guideline, updated and revised [24].

## 2 Case presentation

A 7-year-old girl who had no known past medical issues came to the outpatient department (OPD) in Karachi complaining of a sudden, intense, and piercing stomachache and loss of appetite. The pain was sporadic, lasting around an hour each time, and it was exacerbated after ingestion of food, but it got better after taking analgesics.

She was a socially engaged kindergartener, 105 cm tall and weighing 15 kg. On General physical examination, her vitals were stable, but she appeared pale and irritable with patchy alopecia and halitosis. Her abdomen was not enlarged with normal bowel sounds, but there was a firm, non-tender, palpable epigastric mass. The remainder of the examination revealed nothing noteworthy. According to her mother, she had a history of eating her scalp hair.

## 3 Investigations

**Fig. 1:**
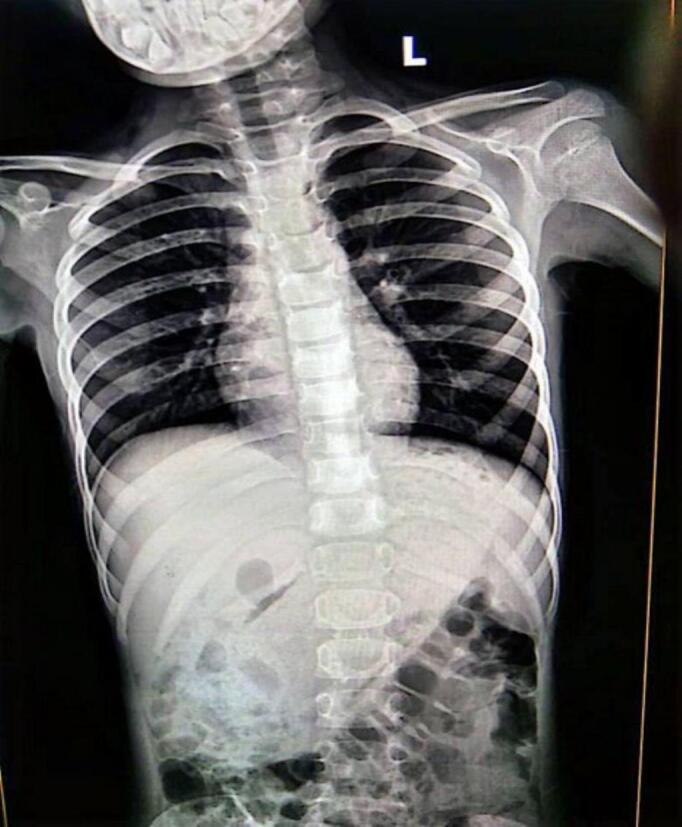
This is an AP view X-ray of the chest and abdomen showing clear lung fields, a normal cardiac silhouette, and no bony deformity There is a well-defined intragastric soft tissue density mass is visible, projecting over the left upper quadrant and mid-abdomen with mottled lucencies, consistent with a trichobezoar. No evidence of perforation or bowel obstruction is observed.

**Fig. 2:**
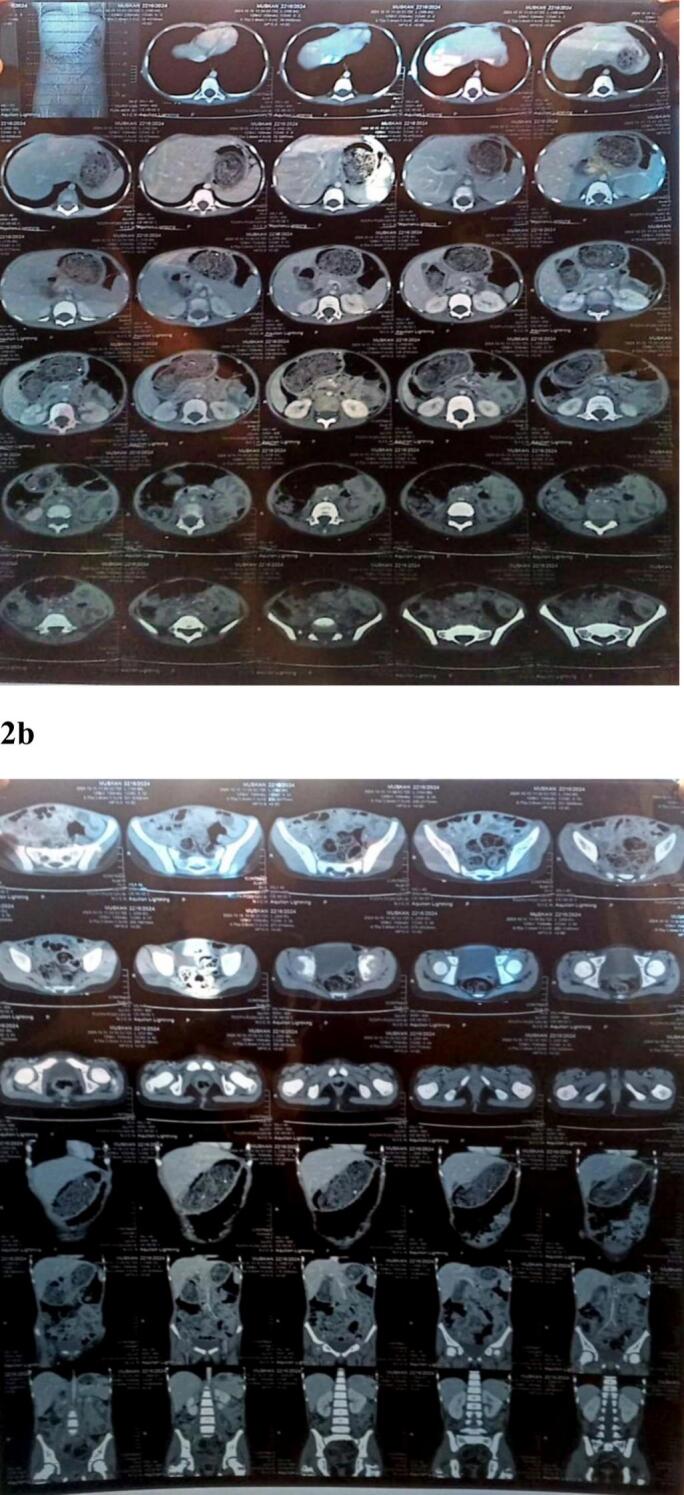
a and b. This is a contrast-enhanced CT scan (axial and coronal views) that revealed a large gastric trichobezoar with extension into the duodenum and proximal jejunum, consistent with Rapunzel syndrome. Mild proximal small bowel dilatation suggested jejunojejunal intussusception with a lead point of mesenteric lymphadenopathy, with no signs of perforation or ischemia.

With a mean corpuscular volume (MCV) of 64.2 FL and a hemoglobin (Hb) level of 10.2 g/dL, laboratory testing demonstrated microcytic anemia. The results of the blood cultures were negative. A large intragastric mass measuring approximately 15 × 7 cm was indicated by a radio-opaque mass inside the stomach that was displacing adjacent bowel loops inferiorly on an abdominal X-ray (Fig. 1). The results of the abdominal ultrasound showed no focal masses or abscesses, along with mild hepatomegaly and a uniform texture. Nonetheless, several enlarged mesenteric lymph nodes were observed, which most likely indicated inflammatory alterations. A computed tomography (CT) scan demonstrated an intra-luminal heterogeneous structure with entrapped air locules as well as scattered hyper-densities giving a mottled appearance extending from the fundus right up to the antrum through the non-dilated C-loop of the duodenum up to the duodenojejunal junction. In the duodenum and jejunum, it appears to be surrounded by fluid. Evidence of Jejunojejunal intussusception was further revealed with mesenteric lymphadenopathy serving as the primary finding. These findings are illustrated in Figs. 2a and b. Pneumoperitoneum and intestinal blockage, which are indicative of gastric bezoar/Rapunzel Syndrome, were not present. Endoscopy was not performed, and the diagnosis of trichobezoar/Rapunzel syndrome was made on the results of the CT scan.

## 4 Surgical treatment

**Fig. 3:**
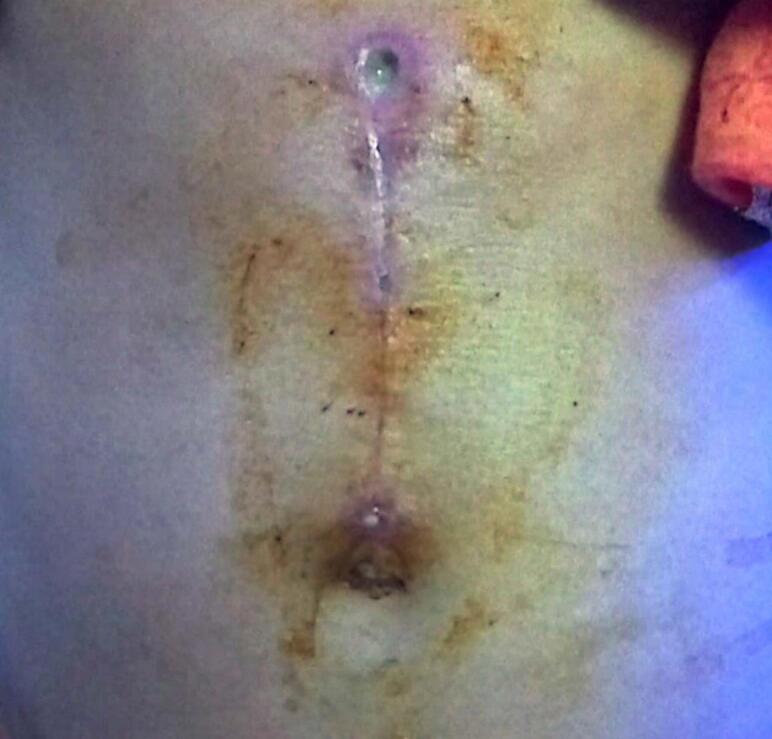
This image showing post-surgical infected scar of the patient.

**Fig. 4:**
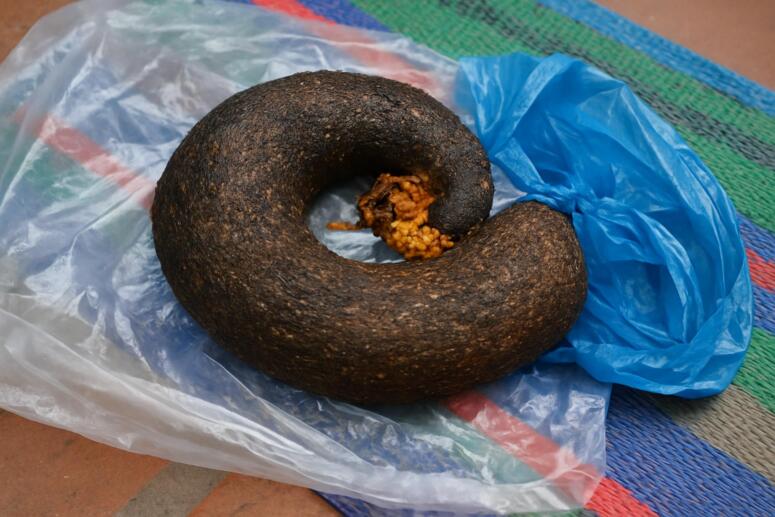
This image showing a gross specimen of a trichobezoar measuring15cm x 7 cm, extracted via laparotomy.

A laparotomy with gastrotomy was scheduled following the results of the CT scan and the large size of the mass. A large midline incision under the xiphisternum up to the umbilicus was made to facilitate the complete removal of the trichobezoar via gastrotomy (Fig. 3). During the procedure, it was found that the whole stomach up to the first part of the duodenum was fully dilated and a large oblique mass measuring approximately 15 × 7 cm filled up the stomach and extended till the second part of the duodenum, supporting the diagnosis of Rapunzel syndrome (Fig. 4). A 5 cm gastrostomy was done on the anterior wall of the stomach, and the mass was removed in one piece. During the procedure, she was administered cefotaxime 1 g, and there were no complications intraoperatively.

## 5 Follow-Up and post-operative complications

The patient was discharged after 4 days of surgery with a successful recovery. Trichophagia, anxiety, and trichotillomania were identified by psychiatric evaluation. To prevent recurrence, she was referred to the psychiatric department.

After a few days, she revisited OPD since she developed an infected surgical wound with purulent discharge (Fig. 3), with pus culture positive with growth of *Escherichia coli* (*E. coli*) and Candida species isolated. To treat this, she was prescribed Amikacin (115 mg) twice a day for seven days in addition to strict wound care. To maintain her nutritional needs, she was prescribed Vidalyn-L syrup for a month. After seven days of treatment, her wound was cured.

## 6 Discussion

Vaughan et al. initially reported Rapunzel Syndrome, a rare kind of obstructive trichobezoar, in 1968 [8]. It is made up of swallowed hair that mostly builds up in the stomach but can also spread into the small intestine and, in certain situations, the colon as it grows. The Grimm Brothers' 1812 tale about a long-haired maiden is where the term comes from. Furthermore, an autopsy in 1779 revealed the earliest known instance of a bezoar in a human [9]. Trichobezoars range in size from tiny (2 × 2 cm) to enormous (30 × 15 × 10 cm). Given its dimensions of 15 × 7 cm, the trichobezoar in this instance belongs to the large trichobezoars [10]. Mostly, Bezoars remain in the stomach, but when they extend into the intestines, they are classified as Rapunzel Syndrome.

A rare gastrointestinal condition called bezoars is brought on by a buildup of undigested particles. Although they can form anywhere in the digestive system, the stomach is where they most commonly appear. In particular, trichomes are made up of undigested hair that has oxidized from exposure to stomach acid, giving it a shiny, black appearance. Trichophagia and trichotillomania are exacerbated by neuropsychiatric diseases, such as anxiety, stress, and mood disorders, which are frequently associated with this condition, which is primarily seen in females [7]. Continuous monitoring and follow-up are essential in preventing recurrence because of the psychiatric implications [11]. It has been estimated that about 30 % of people with trichotillomania have trichophagia, although only 1 % of those who have trichophagia develop a trichobezoar [12]. Typical symptoms include nausea, vomiting, acute abdominal pain, early satiety, weight loss, and epigastric discomfort.

Rapunzel syndrome is rare in children under 10 years of age, making this patient's presentation noteworthy. In addition to the trichobezoar, the occurrence of jejunojejunal intussusception represents an unusual and rarely reported complication. Furthermore, the postoperative wound infection with Candida species constitutes an uncommon co-infection in such cases, adding to the clinical uniqueness and educational value of this report.

Since this condition frequently exhibits no symptoms in its early stages, diagnosing it can be difficult. Early detection becomes challenging since symptoms become less precise as the hair mass grows. A thorough patient history and a high index of suspicion are necessary for diagnosis. In around 85 % of cases, a physical examination reveals a well-defined, hard, and tender mass, and laboratory testing often reveals iron deficiency anemia. Serious problems may arise from a delayed diagnosis. Intestinal blockage, perforation, intussusception, hemorrhage, peritonitis, pancreatitis, and cholangitis are all possible outcomes of large trichobezoars.

The diagnosis of stomach trichobezoars is not very successful with conventional X-ray and ultrasound imaging. Rather, the optimal diagnostic methods are upper gastrointestinal (GI) endoscopy and Computed Tomography (CT) scans [13].

The best treatment for trichobezoars is the complete removal of the hair mass. Although several surgical techniques exist, laparotomy remains the most effective and widely preferred approach.

## 7 Challenges

Owing to this condition's rarity and the patient's financial situation, the diagnosis of trichobezoar in our patient presented with some obstacles, which led to misdiagnosis initially and then delayed diagnosis. Our patient's treatment first commenced in a public hospital, where, due to a lack of equipment and expertise, she was misdiagnosed with a case of hepatomegaly and consequently received antibiotic treatment. Upon unsatisfactory outcomes from treatment and consistent complaints about what she originally presented with, she then moved from her primary hospital to a secondary hospital. From there, she was then referred to a tertiary care hospital to get her CT scan done, but due patient's low socioeconomic background, an MRI could not be performed due to financial constraints. However, a CT scan and then further tests altogether aided the correct diagnosis of trichobezoar, followed by an operation, which achieved the resolution of the mystery of this unusual case.

## 8 Literature review

According to a retrospective study by Liang and colleagues [14], the median patient age for Rapunzel syndrome is 9.1 years, and the condition is most commonly seen in youngsters. Anorexia, nausea, vomiting, anemia, and abdominal discomfort are the clinical symptoms that are most described [14]. A palpable, indentable mass in the epigastric area and a positive Lamerton's sign are also typical findings in afflicted patients [15]. According to the literature, trichotillomania and trichophagia are the main underlying causes. To control the psychological reasons causing this disease, psychiatric follow-up is frequently advised.

Atypical Rapunzel syndrome instances and sequelae are also presented in this review; these should be considered when assessing young female patients with linked psychological illnesses. Because these appearances are uncommon, it is essential to recognize them to avoid potentially fatal outcomes. For example, a 37-year-old woman who had been asymptomatic at first was diagnosed with Rapunzel syndrome exacerbated with pancreatitis and cholangitis [16]. Rapunzel syndrome, resulting in peritonitis, was the subject of another recorded case [17]. There have also been reports of fungal peritonitis and fungal balls following surgery [18]. Additionally, there has been a case of enteral perforation peritonitis with imminent septic shock [19]. Treatment plans in these situations included measures like fluid resuscitation, urinary catheterization, and antibiotic medication before surgery, in addition to addressing the gastric trichobezoar and associated comorbidities. However, in our case, preoperative care was not required, and Amikacin was administered postoperatively due to surgical wound discharge.

A case study [20] detailed the death of a four-year-old girl who experienced severe gastrointestinal blockage as a result of three enormous trichobezoars, even though fatalities from Rapunzel syndrome are uncommon in individuals older than six. After consuming food, her condition deteriorated, leading to repeated vomiting and ultimately, death. On the other hand, our situation resulted in successful surgical procedures and subsequent psychiatric counseling. A wound protector was utilized during surgery in another recorded instance [21] of Rapunzel syndrome recurrence to prevent harm to the surgical site from nine years prior. This patient sought medical assistance for psychological discomfort rather than physical concerns, and they showed no signs of nausea or changes in their bowel habits.

A case of Rapunzel syndrome with severe hypoproteinemia—with blood protein levels as low as 2.5 g/dL—was reported in another study [22], but our laboratory tests did not include serum protein levels. Abdominal blockage, abdominal TB, and paralytic ileus were among the differential diagnoses in that instance. Even though our patient had a history of trichophagia, some instances also showed pica, as the four-year-old girl in another report [15]. Additionally, another recorded case [23] presented with severe anemia, abnormal liver function tests, hematemesis, localized peritonitis, a palpable epigastric mass, and coagulopathy [22], although our case did not exhibit any abnormalities in liver function. Table 1 demonstrates the summary and key points of the presented cases.

**Table 1:** Summary of literature review on Rapunzel Syndrome.

Author(s)	Year	Study type	No. of patients	Age	Key findings
Liang et al. [14]	2024	Retrospective study	10	Median patient age is 9.1 years	All patients were female with most common clinical symptoms upper abdominal mass (90 %), abdominal pain (80 %), and nausea and vomiting (50 %). All patients underwent surgical treatment, and recovered well.
Lamerton's Sign (Pokhrel et al.) [15]	2025	Case report	1	4-year-old	female patient presented with pain and a lump over the epigastric region with an episode of vomiting, early satiety, and a history of ingestion of hair. CT scan suggesting bezoars extending up to the duodenum
Vellaisamy et al. [16]	2020	Case report	1	37-year-old	Rare case of a 37-year-old woman with Rapunzel syndrome complicated by acute cholangitis and pancreatitis
Belhadj et al. [17]	2023	Case report	1	19-year-old	female hairdresser with a history of trichophagia and have abdominal pain and episodes of vomiting. Diagnosed with Rapunzel syndrome accompanied by pneumoperitoneum and intraperitoneal effusion.
Sotoudeh et al. [18]	2019	Case report	1	7-year-old	Malnourished female with a 1-year history of endoscopy-proven trichobezoars reported fungal peritonitis and fungus balls as postoperative complications.
Uttam et al. [19]	2023	Case report	1	15-year-old	Female presented with signs and symptoms of enteric perforation with a history of trichotillomania and trichophagia.
Piras et al. [20]	2023	Case report	1	4-year-old	Reported death of a girl due to GI obstruction by three massive trichobezoars.
Obinwa et al. [21]	2017	Case report	1	25-year-old	Recurrence case in a female patient where a wound protector was used to avoid damage to prior surgical site.
Kumar et al. [22]	2019	Case report and literature review	1	19-year-old	Female patient with severe hypoproteinemia (2.5 g/dL); differential diagnosis included TB and paralytic ileus.
Agarwal et al. [23]	2025	Case report	1	19-year-old	Female patient with Rapunzel syndrome with severe anemia, abnormal liver function, hematemesis, coagulopathy, and localized peritonitis.

## 9 Conclusion

A rare but possibly harmful medical illness is trichobezoars, or Rapunzel syndrome. The risk factors that contribute to their development must be identified by physicians. A comprehensive patient history, clinical examination, and prompt investigations are necessary for the diagnosis. The most effective treatment for this problem is still laparotomy. Psychiatric surveillance ought to be a crucial component of patient care in order to stop recurrence.

## Abbreviations

GI: Gastrointestinal
OPD: Outpatient Department
MCV: Mean Corpuscular Volume
Hb: Hemoglobin
CT: Computed Tomography
AP: Anteroposterior
*E. coli*: *Escherichia coli*
TB: Tuberculosis

## Consent

Written informed consent was obtained from the patient's parents/legal guardian for publication and any accompanying images. A copy of the written consent is available for review by the Editor-in-Chief of this journal on request.

## Ethical approval

This case report was conducted by institutional ethical standards. Ethical approval was obtained from the local ethics review board. All identifying information has been anonymized to maintain patient confidentiality.

## Funding

The authors received no extramural funding for this case report.

## Author contribution

Umer Iqbal did the conceptualization of the manuscript; Umer Iqbal, Sara Jawaid, Sarah Sohail, Syed Ali Arsal and Rehman Asif write the manuscript. Syed Ali Arsal and Umer Iqbal did the editing. Syed Ali Arsal and Inibehe Ime Okon did the supervision during the whole process. We, the undersigned, declare that this manuscript is original, unpublished, and not under consideration elsewhere. We confirm that the manuscript has been read and approved by all named authors and that there are no other persons who satisfied the criteria for authorship but are not listed. We further confirm that the order of authors listed in the manuscript has been approved by all of us. We understand that the senior and corresponding author (Inibehe Ime Okon) is the sole contact for the editorial process and did the supervision during the whole process. He is responsible for communicating with the other authors about progress, submissions of revisions, and final approval of proofs.

## Guarantor

Inibehe Ime Okon.

## Research registration number

Not applicable.

## Conflict of interest statement

The author(s) declare no potential conflicts of interest concerning the research, authorship, and publication of this article.
